# Development of a New Bioequivalent Omeprazole Product

**DOI:** 10.3390/medicina60030427

**Published:** 2024-03-02

**Authors:** Gulzina Kumisbek, David Vetchý, Arshyn Kadyrbay

**Affiliations:** 1 Department of Pharmaceutical Technology, Faculty of Pharmacy, Masaryk University, 612 00 Brno, Czech Republic; kgulzzina@gmail.com; 2 Viva Pharm, Almaty 050060, Kazakhstan; karshyn@internet.ru

**Keywords:** omeprazole, bioequivalence study, stability study, enteric coating, pellets, dissolution, industrial development

## Abstract

*Background and Objectives:* The enteric form of omeprazole is one of the most commonly prescribed medications. Similarly to Europe, Kazakhstan relies on the localization of pharmaceutical drug production as one of its primary strategies to ensure that its population has access to affordable and good-quality medicines. This study comprehensively describes the technologically available development of bioequivalent delayed-release omeprazole. *Materials and Methods:* Various regimes and technological parameters were tested on laboratory- and production-scale equipment to establish a technical process where a functional and gastro-protective layer is essential. According to the ICH guidance on stability testing and Kazakhstan local rules, stability studies were conducted under conditions appropriate for climate zone II. The comparison of the rate and extent of absorption with subsequent assessment of the bioequivalence of the generic and reference drugs after a single dose of each drug at a dose of 40 mg was performed. *Results:* The quantitative and qualitative composition and technology of producing a new generic enteric form of omeprazole in capsules were developed and implemented at the manufacturing site of solid forms. Dissolution profiles in media with pH 1.2 and 6.8 were proven. During the accelerated six-month and long-term twelve-month studies, the developed formulation in both packaging materials at each control point passed the average weight and mass uniformity test, dissolution test, acid-resistance stage test, buffer stage test, impurity assay, and microbiological purity test and met all the specification criteria. A bioequivalence study in 24 healthy volunteers compared against the innovative drug showed the bioequivalency of the new generic system. The obtained values from the test and reference products were 1321 ± 249.0 ng/mL and 1274 ± 233 ng/mL for C_max_, 4521 ± 841 ng·h /mL and 4371 ± 695 ng·h /mL for AUC_0-t_, and 4636 ± 814 ng·h /mL and 4502 ± 640 ng·h /mL for AUC_0-∞_. *Conclusions:* Using affordable technologies, a bioequivalent generic delayed-release formulation of 20 and 40 mg omeprazole has been developed.

## 1. Introduction

Peptic ulcer disease (PU) is a chronic, recurrent acid-dependent disease characterized by alternating periods of exacerbation and remission. It is considered the most common disease of the digestive system, affecting 10–15% of the world’s population. The main component of gastric juice is hydrochloric acid. When secreted at levels above normal, it has a pathological effect and is the main factor in the pathogenesis of ulcers. This pathology shows defects (ulcers) in the wall of the stomach and duodenum. In contrast to erosive changes in the mucosa, an ulcer affects the mucous membrane as well as the submucosal layer [1,2,3].

Currently, proton pump inhibitors (PPIs) are the primary drugs used to treat acid-related diseases of the upper gastrointestinal tract due to their highly selective inhibitory effect on the acid-forming function of the stomach. The primary drug from the PPI group is omeprazole, which was synthesized in 1979 after the discovery of a unique enzyme, K^+^-stimulated ATPase, in parietal cells during experiments on the gastric mucosa [2,4,5]. Omeprazole, as an agent that reduces gastric acid secretion, is prescribed for the treatment of the following diseases: duodenal ulcer; gastric ulcer; NSAID-associated (non-steroidal anti-inflammatory drugs) gastric and duodenal ulcers; reflux esophagitis; symptomatic gastroesophageal reflux disease (GERD), i.e., heartburn and regurgitation; Zollinger–Ellison syndrome (pathological hypersecretory condition); Helicobacter pylori (H. pylori) [3]. In 2022, omeprazole was found to be the second most commonly prescribed chemical substance in England, with the drug listed on approximately 35 million prescriptions [6]. According to the Drug Usage Statistics for 2021 in the USA, omeprazole was ranked as the ninth top drug in the market, with an estimated number of 54.5 million prescriptions [7].

In the Republic of Kazakhstan, drugs for patients with acidity disorders are the eighth most often prescribed according to the ATC (Anatomical Therapeutic Chemical) classification system. Omeprazole is among the top 15 main molecules (active ingredients) of the general prescription market and ranks 13th in terms of importance (Vi-ORTIS data). PPIs account for 75.3% in monetary terms and 89.6% in volume terms of the total market for medicines used to treat acid-related disorders. Omeprazole ranks first among the top ten PPI market leaders in terms of the number of packages sold, accounting for 71.66% of the total PPI market in volume terms, and in monetary terms, it comes to 43.56% of the total PPI market [8].

The enteric form is the most common form of omeprazole oral dosage formulations. Since omeprazole is a pro-drug that accumulates in the acidic space of the parietal cell and is converted into an active state there, it has the characteristics of a weak base, so it remains stable in neutral pH but degrades rapidly in an acidic environment. Therefore, to reach the small intestine where it is absorbed, omeprazole must be protected from gastric acid when administered orally. These characteristics are a challenge for drug developers in the search for optimal delivery of the pharmacologically active substance to the desired site of absorption or activation [8]. Ten products are marketed in Kazakhstan: a powder for suspension for oral administration (India), one lyophilizate for intravenous administration (India), and ten enteric capsules (Belarus, India, Spain, Slovenia, Russia). Only one of these is manufactured in Kazakhstan. The presented capsules contain enteric-coated micropellets, pellets, granules, and microgranules [8,9].

There have been several inventions aimed at producing enteric-coated pellets of PPIs. These pellets consist of an empty pellet core, a drug-loaded layer, isolating layers I and II, and an enteric-coated layer coated using a fluidized bed coater [10,11,12]. Against this background, the development of an enteric form of omeprazole in Kazakhstan has been of great interest. However, the coating of pellets or granules is a complex process that requires a high level of technology. The technical capacity to produce such products on an industrial scale is not available in all manufacturing locations. This article describes the technologically feasible development of a bioequivalent extended-release generic product containing 20 mg and 40 mg of omeprazole, focusing on an in vivo bioequivalence study. 

## 2. Materials and Methods

### 2.1. Materials

The following materials were used in the study: omeprazole (Hetero Labs, Hyderabad, India) Active Pharmaceutical Ingredient (API), lactose monohydrate Supertab 22AN (Glentnam Life Sciences Ltd., Corsham, UK), sodium lauryl sulfate (RNDr Kulich Pharma s.r.o., Hradec Králové, Czech Republic), disodium hydrogen phosphate dodecahydrate cryst. EMPROVE^®^ (Merck KGaA, Darmstadt, Germany), hydroxypropylmethylcellulose (Tailopur 603), hydroxypropyl cellulose Klucel EF Pharm (Ashland Industries Europe GmbH, Schaffhausen, Switzerland), pellets from microcrystalline cellulose (MCC) Cellets^®^700 (Process center GmbH&Co.Kg, Dresden, Germany), polyethylene glycol 400 (Applichem GmbH, Darmstadt, Germany), methacrylic acid–ethyl acrylate copolymer Eudragit^®^ L30-D55 (Evonic Nutrition and Care GmbH, Darmstadt, Germany), hard gelatin capsules size 0 (Capsugel, Bornem, Belgium), PlasAcryl^®^ HTP20 (Emerson Resources Ink., Norristown, PA, USA), titanium dioxide (Venator Germany GmbH, Krefeld, Germany), talc (Imerys Talc Italy S.p.A, Porte, Italy). All ingredients were of pharmaceutical production grade as described in the European Pharmacopoeia, and are widely used in preparations for oral use at concentrations not exceeding the recommended limits. Losec^®^, enteric capsules, 20 mg, AstraZeneca AB, Sweden, was used as the reference drug.

### 2.2. Preparation of Omeprazole Pellets

In order to define the drug’s qualitative and quantitative composition and production technology, several experimental batches were produced in laboratory and pilot sizes at the Department of Pharmaceutical Technology, Faculty of Pharmacy, Masaryk University Brno, Czech Republic (the University). Quality control analytical methods and dissolution profile tests were determined and performed accordingly during the experiments. The developed composition and production technology were tested and improved on a pharmaceutical production scale at Viva Pharm, Almaty, Kazakhstan (Viva Pharm). 

The sizes of the laboratory and pilot batches produced at the University ranged from 0.210 kg to 2.1 kg. At Viva Pharm, the pilot batches were manufactured on a production scale with a batch size of approximately 10.9 kg. The batch intended for the bioequivalence study was 45.68 kg size/200,000 capsules (20 mg)/100,000 capsules (40 mg). The pellets containing omeprazole were prepared in three main steps: I, active coating; II, protective coating; and III, enteric coating. 

#### 2.2.1. Active Coating

In this step, omeprazole suspensions from two suspensions, IA and IB, were coated on inactive microcrystalline cellulose cores. 

Suspension IA was prepared by mixing the following ingredients: omeprazole, lactose monohydrate, sodium lauryl sulfate, and di-sodium phosphate dodecahydrate in purified water.

The second, IB, a solution of hydroxypropylmethylcellulose (HPMC) and hydroxypropylcellulose (HPC), was prepared by dispersing the components in purified water heated to 80 °C followed by slow cooling at room temperature for 3 h. Parts IA and IB were then mixed and sprayed onto inactive cores in fluidized bed coaters: Medipore, Czech Republic, for laboratory-scale batches, and Aeromatic-Fielder AG, Switzerland, FBD Bosch pilotlab L, Germany, for pilot-scale batches. The following equipment was used at Viva Pharm for pilot- and production-scale batches: FBD Bosch pilotlab L; calibrator, Frewitt ConiWitt-150; shelf dryer, Hansung HS-DO-1.4; conical granulator, Keno KZ SKZ-180; moisture analyzer, KERN DS8K0.5; overhead mixer Jeio Tech Lab MSD 0420; mixers, Hansung HS-DM 200 and UPT HLS-50H.

#### 2.2.2. Protective Coating

The protective coating solution (Solution II) was prepared by gradually adding pre-weighed hydroxypropylmethylcellulose to the purified water with continuous stirring until it was completely dissolved and homogenized. After that, Solution II was sprayed onto the active cores in fluidized bed coaters using the same parameters as used in step I, active coating.

#### 2.2.3. Enteric Coating

First, a titanium dioxide suspension was prepared by adding a weighed amount of titanium dioxide to the purified water and mixing it until it was homogeneous. In order to obtain the dispersion for enteric coating III, Eudragit L30-D55, PlasAcryl, and the prepared titanium dioxide suspension were added to weighed purified water with continuous stirring using an overhead stirrer for at least 120 min. 

In all three stages of the coating process, 1.2 mm diameter nozzles were used, with a spray air pressure of 1–3 bar. After the coated pellets were dried at the end of all three steps, the prepared product was unloaded using the Frewitt ConiWitt-150 vacuum system and calibrator without the installation of a sieve or a knife. The coated pellets were sieved through 1.5 mm sieves to separate the undesired agglomerates. The prepared pellets were mixed with talc and then filled into hard gelatin capsules in the Zanasi 40E capsule-filling machine (IMA S.p.A., Bologna, Italy). The capsule sizes were No. 2 for the strength of 20 mg and No. 0 for the strength of 40 mg.

The omeprazole content of one capsule was determined using high-performance liquid chromatography (HPLC) with an ultraviolet (UV) detector. 

### 2.3. Dissolution Study

Dissolution tests were performed according to the USP monograph by using the “Distek 2500” dissolution tester (USP type II apparatus with a paddle) at a stirrer rotation speed of 100 rpm and the dissolution medium temperature of 37.0 ± 0.5 °C. An acid resistance test was performed in a dissolution medium of 500 ml of 0.1 M hydrochloric acid (HCl) pH 1.2 for 2 h. After two hours, for the dissolution buffer stage, 400 mL of 0.235 M dibasic sodium phosphate was added to the 500 mL of 0.1 M hydrochloric acid medium in the vessel and adjusted, if necessary, with 2 M hydrochloric acid or 2 M sodium hydroxide to the pH of 6.8 ± 0.05. Samples were taken at 10, 15, 20, 30, and 45 min. The amount of omeprazole released was determined by HPLC. The original drug Losec and the prepared samples were compared using similarity factors f_2_. The dissolution kinetics of drugs are considered similar when the similarity factor f_2_ is equal to or greater than 50 [13]. 

### 2.4. Stability Study

Stability studies were conducted under conditions appropriate for climate zone II according to the ICH guidelines for stability testing and local regulations of Kazakhstan [14,15]. Qualified climate chambers were used for long-term studies at the temperature of 25 °C ± 2 °C and the relative humidity (RH) of 60% RH ± 5% RH and for accelerated studies with the regime of 40 °C ± 2 °C/75% and RH ± 5% RH.

### 2.5. Bioequivalence Study

The objective was to compare the rate and extent of absorption followed by a bioequivalence assessment of the generic and reference (originator) drugs after a single dose of each drug at a dose of 40 mg.

#### 2.5.1. Study Products

Two finished drugs of omeprazole were compared:

Reference formulation (R): Losec^®^, enteric capsules, 20 mg (2 capsules), No. 14, AstraZeneca AB, Södertälje, Sweden (batch number 3070122, expiration date May 2024).

Test formulation (T): omeprazole Viva Pharm, enteric capsules, 40 mg, No. 30, Viva Pharm LLP, Almaty, Kazakhstan (batch number 00122, expiration date November 2024).

#### 2.5.2. Ethics Compliance and Subjects

The study was conducted in accordance with the conclusion of the local commission on bioethics issues of the Center for Nuclear Medicine and Oncology of the Health Department of the Abai region (No 1 dated 26 January 2023), and the clinical study protocol (the protocol) was approved by the National Center of Medical Drug Expertise of the Ministry of Health of Kazakhstan (No BE-Omep-2022/02, version 4, dated 6 April 2023) in compliance with the principles of the Declaration of Helsinki (Seoul, 2008), Good Clinical Practice (GCP), and local regulations on bioequivalence study [16]. The clinical part of the study was conducted in the Center for Nuclear Medicine and Oncology of the Health Department of Abai region, Abay region, Semey City, Kutzhanova 3, Kazakhstan, and the analytical part was conducted in the laboratory of pharmacokinetics and metabolomic analysis of the Institute of Translation Medicine and Biotechnology of Sechenov University, 117418, Moscow, Nakhimovsky prospect 45.

Thirty male and female volunteers aged 18–45 participated in the screening. The screening procedure followed the study protocol before the first study period. After obtaining written informed consent from the volunteer, the screening included registration of demographic data, physical examination, medical history, measurement of vital signs (blood pressure, heart rate, respiratory rate, body temperature), assessment of well-being, electrocardiogram, clinical laboratory tests (complete blood count, urinalysis, biochemical parameters, hepatitis B surface antigen, hepatitis C antibody and human immunodeficiency virus antibodies, microreaction, alcohol test, pregnancy test, and urine drug test), and the assessment of applicable inclusion and exclusion criteria. Detailed descriptions of the inclusion and exclusion criteria used in this study and the criteria for subject withdrawal from participating are presented in the supporting material. Six volunteers were excluded after the screening based on the clinical laboratory test results. As a result, 24 healthy volunteers, 16 males and 8 females aged 18–45 years (mean age 29.5 years), were included in the study. All volunteers in the study were continuously monitored and participated until the completion of the study.

#### 2.5.3. Study Design and Safety

The study consisted of screening, two study periods, and a washout period (crossover study design). The duration of the screening period was up to 10 days. Each study period lasted for 13 h, and the washout period was seven days. The total time of the study for one volunteer was no more than 20 days.

At each period of the study, in the morning, after overnight fasting (minimum of eight hours), the volunteers were administered a single dose of 1 capsule with a dosage of 40 mg of the test drug or two capsules containing 20 mg of the reference drug; they swallowed the whole capsule without chewing and drank 250 mL of water. Water consumption was limited to 1 hour before taking the drug and 1 hour after taking the medicine. The first meal was 5 min after taking the medications, and lunch was taken 6 h after the drug administration. A standard diet, including food and beverages, was established for all volunteers during all study periods.

A total of 576 blood samples were collected from 24 volunteers in two phases of the study. Sampling time was at 0.00 (zero sample, blank point, 15 min before drug administration), 0.50, 1.00, 1.50, 2.00, 2.50, 3.00, 4.00, 5.00, 6.00, 8.00, and 12.00 h after the drug administration. After 12 h, the volunteer’s venous catheter was removed. Blood in an amount of 5 mL was collected into heparinized tubes and processed within 20 min. Plasma was separated by centrifugation for 15 min at a speed of at least 3000 rpm/min and then stored at a temperature of −20 °C until it was sent to the pharmacokinetic laboratory.

During the study, according to the schedule (Table 1), appropriate procedures were performed to ensure the safety and well-being of the volunteers, to identify adverse events, and to assess the possibility of further participation of volunteers in the study.

#### 2.5.4. Analytical Method

Omeprazole concentrations were determined by a validated method of HPLC with tandem mass spectrometry on a Waters Acquity UPLC device with a Xevo TQS micro tandem quadrupole mass spectrometric detector, Waters Corporation, Milford, MA, USA (HPLC-MS/MS), and an Acquity UPLC BEH C18 analytical and pre-analytical column (2.1 × 50 μm; 1.7 μm), Waters Corporation, USA, using mobile phase A (0.1% solution of formic acid in water) and mobile phase B (0.1% formic acid in acetonitrile) [17,18,19]. A gradient elution program was applied with a 99:1 ratio of mobile phase A to B, which reversed during the analysis. The following software was used for validation: results processing software package Target Lynx Application Manager from Waters Corporation, USA; for statistical processing of the analysis results, the Jamovi 2.01 program (R 4.0 package), Microsoft Excel, or Minitab 16 from Minitab Inc., State College, PA, USA, was used. 

The external standard method, linear regression, least square regression, and a 25.0–2000 ng/mL calibration range for omeprazole were used to detect omeprazole concentration. Calibration standards and quality control (QC) samples were prepared independently using individually prepared solutions. All samples (calibration standards, QC samples, and test samples) were processed as a single batch of samples.

One series consisted of samples that were prepared simultaneously, i.e., processed sequentially without interruption by the same laboratory operator using the same reagents and under the same conditions. The study attempted to ensure that all samples from a single study subject were analyzed in a single analytical run. In this case, QC samples were distributed throughout the analytical cycle to ensure control of the accuracy and precision of the entire process [17,18].

#### 2.5.5. Pharmacokinetic and Statistical Analysis

Descriptive statistics were calculated and presented for the main and additional pharmacokinetic (PK) parameters of omeprazole (N, median, arithmetic mean, geometric mean, minimum and maximum values, standard deviation, coefficient of variation). For logarithmically transformed PK parameters and omeprazole Cmax and AUC_0-t_, a bioequivalence assessment was carried out by the analysis of variance (ANOVA) in a mixed-effects model and the analysis of LSM (T/R) ratios of in-transformed PK parameters. Drugs were considered bioequivalent if the 90% confidence interval for the ratio of the geometric mean values of Cmax and AUC_0-t_ of omeprazole was 80–125%. The analysis of variance was used to test the hypothesis of the statistical significance of the contribution of each of these factors to the observed variability. The estimate of residual variation obtained using variance analysis was used to calculate the 90% confidence interval for log-transformed values of the corresponding pharmacokinetic parameter. When selecting samples, the volunteers were not divided into subgroups; therefore, a modified statistical model was not required. The main parameters considered were the logarithmically transformed parameters AUC_0→t_ (area under the curve from 0 to 12 h), C_max_ (maximum value of the drug concentration in the blood), and the C_max_/AUC_0-t ratio_. The statistical analysis included the following procedures: calculation of basic and ordinal statistics, analysis of variance, graphical methods, computation of confidence intervals, calculation and analysis of bioavailability ratios, and testing of interval tests. The differences between the mean values of pharmacokinetic parameters ln(C_max_), ln(AUC_0-t_), and ln(C_max_/AUC_0-t_) were assessed at a 5% significance level, by analyzing the sum-of-squares (SS), degrees of freedom (DFs), mean squares (MSs), F ratio, and P-value using parametric analysis of ANOVA. The contributions of the factors “drug” (differences between drugs), “subject sequence” (differences between volunteers, interindividual differences), “sequence” (variability due to the sequence of drug administration), and “period” (variability due to the study design) were examined as sources of variation [19].

## 3. Results

### 3.1. Development of the Composition and the Production Technology of the Drug

The quantitative content of the active substance and the dissolution profile test results defined necessary corrections in the formulation and technology to achieve a finished product meeting the quality specification requirements. The omeprazole content in one capsule had to be from 18.0 to 22.0 mg/cap (for the dosage of 20 mg) and from 36.0 to 44.0 mg/cap (for the dosage of 40 mg) and was detected by the HPLC assay method. Another critical test, the dissolution study, determined an amount of omeprazole released at different pH conditions, showing how the formulation was protected in the acid-resistance stage (1.2 pH) and was sufficient at the buffer stage (6.8 pH). Thereby, the qualitative and quantitative composition of the pellets, in particular, the concentration of the coating solutions/dispersions and the technological parameters, was slightly adjusted to the characteristics of the equipment and based on the results of the tests for the quantitative content of the active ingredient and the dissolution profile tests. This was carried out in the course of multiple experiments in the production of laboratory and pilot sizes of batches. The technological parameters of the production processes were also amended according to the type and technical features of the equipment at the Viva Pharm site.

The formulation adjustments included an increase in the quantity of MCC pellets per capsule from 60.45% to 62.44% and a change in the size from 500 to 700 µm in order to obtain a thinner API layer. Furthermore, polyethylene glycol was replaced with PlasAcryl^®^ HTP20 to improve product efficiency and reduce the complexity of technological operations. The content of hydroxypropyl cellulose was reduced from 2.1% to 0.44% until a sufficient result of API release was reached. Hydroxypropylmethylcellulose (from 7.97% to 8.67%), Eudragit L30-D55 (from 5.76% to 8.75%), and PlasAcryl (from 1.07% to 1.49%) were added due to the increase in the core area and to achieve better protective and enteric layers. Titanium dioxide (0.44%) was added to the composition for whitening, and talc (0.18%) for better lubrication. Finally, Composition 4 was prepared and used to manufacture the production batches for stability and bioequivalence studies (Table 2).

It was found that the quality of the product was very sensitive and also dependent on the technical parameters of the coating process. The intermediate product reacted differently to temperature fluctuation, air pressure, inlet volume, and other conditions.

The following process parameters were found to be the most appropriate: The cores were preheated to 42 °C in the active coating process and kept at 34–37 °C. After the layering was complete, the active beads were dried at the product temperature of 40 °C for 30 min. The inlet air temperature was kept within the range of 62–70 °C. For the protective coating process, the cores were preheated at the product temperature of 42 °C, coated at 38–42 °C, and dried at 40 °C for 30 min. For both coating processes, the inlet air temperature range of 62–70 °C was used.

In the enteric coating step, the fluidized bed coater container was preheated at 40–42 °C. The layering process was started at a stable incoming air temperature of 40 °C for 30 min and a container temperature of 30 °C. During the coating of the cores, the temperature of the product was kept at 25–28 °C, and for drying, at 32 °C. The inlet air temperature was maintained at 40–47 °C.

### 3.2. Dissolution Study

**Figure 1:**
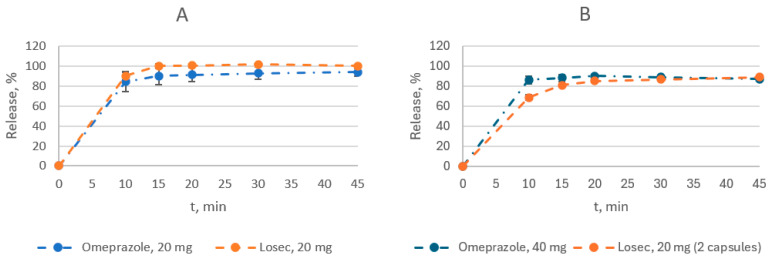
Comparison of dissolution profiles of the test drug and the reference drug Losec^®^ at pH 6.8: (**A**)—20 mg dose; (**B**)—40 mg dose.

As for the dissolution of omeprazole, 20 and 40 mg enteric capsules were compared with the reference drug Losec^®^, 20 mg enteric capsules (Figure 1, Tables S1 and S2).

The dissolution was performed at pH 1.2 for 2 h, followed by 45 min at pH 6.8. The amount of omeprazole released at pH 1.2 was evaluated at a single point in 120 min. Both the test and reference drugs met this requirement. The test and reference drugs released from 3.0% to 10.6% and 1.3% to 5.7% of the omeprazole, respectively (Tables S1 and S2). At pH 6.8, 90.8 to 92.3% of omeprazole and 85.9 to 93.8% of the reference and test drugs were released (Figure 1, Tables S1 and S2).

Since both drugs containing 20 mg of omeprazole had a release level of the active substance of more than 85% at 15 min, the similarity coefficient calculation was not carried out. The dissolution profiles of the drugs were considered similar (Table 3, Figure 1). In the case of 40 mg dosage, the similarity coefficient f_2_ was greater than 50, and the dissolution profiles of the drugs were also considered similar (Table S2).

### 3.3. Stability Study

Since the formulation is sensitive to the environment, the following two types of packaging were selected to protect the drug from moisture, air oxygen, gas vapor, and microbiological contamination: 1. a bottle made of high-density polyethylene, tightly sealed with an aluminum membrane, with a child-resistant polypropylene screw cap; 2. aluminum foil blisters (Alu/Alu): cold-forming aluminum foil with oriented polyamide, aluminum, polyvinylchloride (OPA/ALU/PVC), and aluminum foil coated with polyvinyl chloride film.

A series of stability studies were conducted on laboratory-scale experimental batches and approximately ten pilot-scale sets, including the batch intended for the bioequivalence study for each strength and container closure system mentioned above. For both conditions, the study specification included the following tests: average weight and mass uniformity test, dissolution test, acid-resistance stage test, buffer stage test, impurity assay, and microbiological purity test. The evaluated batches passed the accelerated six-month and long-term twelve-month studies, further continuing the assessment (Tables S3 and S4).

### 3.4. Bioequivalence Study

#### 3.4.1. Subjects

An open, randomized, crossover, two-period bioequivalence study (BES) of the generic omeprazole formulation, enteric capsules, 40 mg, was conducted in comparison with the original product Losec^®^, enteric capsules, 20 mg (two capsules), to evaluate the pharmacokinetic parameters and relative bioavailability of the drugs on 24 healthy volunteers.

No adverse events were identified during the BES study, and the volunteers tolerated both drugs well. At the end of the clinical part of the study, biological material in the amount of 576 biosamples in the form of blood plasma was collected and sent for bioanalytical research.

#### 3.4.2. Assessment of PK parameters

Data from 24 volunteers who completed the study according to the protocol were included for the calculation of the pharmacokinetic parameters and statistical analysis (Tables S5–S8). The analysis was carried out based on the assumption of a non-normal distribution of parameters. To calculate 90% confidence intervals, the values of the analyzed indicators were logarithmized, and then an ANOVA was performed using the Certara Phoenix WinNonlin program and EquivTest.

**Figure 2:**
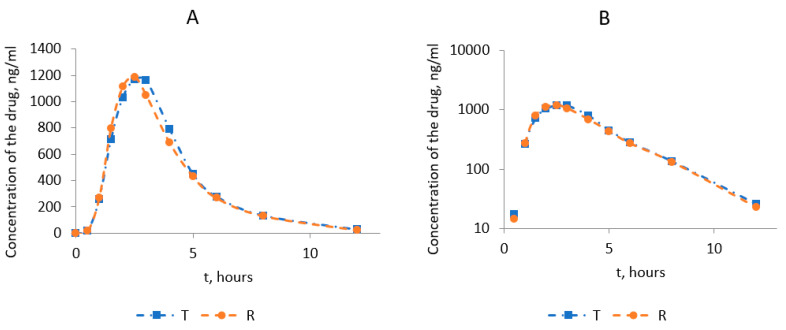
Averaged pharmacokinetic profiles of the test drug and the reference drug Losec^®^: (**A**)—in linear coordinates; (**B**)—in semi-logarithmic coordinates.

Figure 2 shows the average pharmacokinetic profiles of omeprazole after the administration of the test and reference drugs in linear coordinates and semi-logarithmic ones. Standard deviations in the pharmacokinetic profiles are placed in the supporting material (Tables S7 and S8) for better clarity. Most average values of the test and reference drugs are very similar, and it might not be clear which standard deviation belongs to the test and which to the reference drug.

The comparative analysis of the main pharmacokinetic parameters for the test and reference drugs showed that the studied drugs entered the systemic circulation from the gastrointestinal tract at an equal rate (Table 3).

The time to reach the maximum omeprazole concentration (T_max_) averaged 2.54 ± 0.44 h for the test and 2.44 ± 0.43 h for the reference drug. The difference between T_max_ values for the test and the reference averaged 0.1 h. The presented data indicated no statistically significant differences between the studied medicines before the maximum concentration (T_max_) was reached. At the same time, the average maximum concentrations of omeprazole determined in the blood plasma of volunteers (C_max_) were 1321 ± 249.0 ng/mL and 1274 ± 233 ng/mL for the test and the reference product.

The analysis of the primary parameter characterizing the degree and rate of bioavailability of the active substance from the dosage form, and the area under the pharmacokinetic curve (AUC_0-t_), indicated insignificant variability in this parameter. The average AUC_0-t_ values for the test and reference drug were 4521 ± 841 ng/mL·h and 4371 ± 695 ng/mL·h, respectively. The average value for the AUC_0-∞_ parameter was 4636 ± 814 ng/mL·h for the test drug and 4502 ± 640 ng/mL·h for the reference. At the same time, no statistically significant differences were revealed for the compared values. The data in Table 4 show the assessment of the criteria for the sufficiency of the observation period for the study drugs. The values of the ratio AUC_0-t_/AUC_0-∞_ were more than 80%, which indicated that the specified criteria were met.

The analysis of variance of pharmacokinetic parameters did not reveal any significant sources of variation. Testing was also performed to identify outlier individual differences in log-transformed AUC, C_max_, and C_max_/AUC_0-t_ values by plotting standardized differences (mean-centered and standard-deviation-normalized). No outliers were identified when assessing the location of the value indicators for the logarithmically transformed values of AUC, C_max,_ and C_max_/AUC_0-t_. The obtained data do not exceed the permissible limits [−3σ;3σ], are symmetrical relative to the x-axis, and indicate a normal distribution of individual differences.

By assessing the parameters F and p-value of the analysis of variance (ANOVA) for the pharmacokinetic parameters ln(C_max_), ln(AUC_0-t_), and ln(C_max_/AUC_0-t_), it was concluded that the factors “drug”, “period”, “sequence”, and “subject sequence” did not cause any significant differences between the test and reference drugs. The power of the statistical tests for calculations of intra-subject variation was 99.9% for the ln(C_max_) pharmacokinetic parameter, 100.0% for ln(AUC_0→t_), and 100.0% for ln(C_max_/AUC_0→t_). This shows that the power of statistical tests was sufficient (Table 5).

The 90% confidence intervals (CIs) for the μT/μR geometric mean ratios for logarithmically transformed omeprazole pharmacokinetic parameters were as follows:

1. lnC_max_: 95.02–113.15%; μT/μR = 103.69;
2. lnAUC_0-t_: 96.03–110.74%; μT/μR = 103.13;
3. lnC_max_/AUC_0-t_: 96.58–104.67%; μT/μR = 100.54.

The CIs were entirely within the range of values for bioequivalence: 80–125% for lnC_max_ and lnAUC_0-t_ and 75–133% for lnC_max_/AUC_0-t_.

## 4. Discussion

Since omeprazole is unstable in acidic pH conditions, the main challenge was to obtain adequate protective enteric layers on the pellets with the required release profiles. A hydroxypropyl methylcellulose sub-coat was applied to prevent the migration of omeprazole layered on the beads into the enteric coating during its application [20,21]. The experimental part of the laboratory-scale batches solved the most problematic issues related to the size of the cores, the quantity and ratio of excipients, and the preparation methods of the coating solutions. However, during the technology transfer to the production scale, unsatisfactory dissolution test results led to adjusting the quantitative and qualitative composition. Furthermore, it was discovered that the technological parameters of all three coating processes, due to their complexity, directly impact the product’s quality characteristics and can cause even more straightforward consequences, such as sticking of the pellets. On a production scale, reducing the inlet air temperature and consequently the product temperature led to effective results in product quality, process optimization, and loss reduction.

One important quality parameter was the acid resistance of the developed dosage form. According to the USP monograph, no more than 15% of a drug can be released from the dosage form within 120 min at pH 1.2 [13]. Both the test and reference drugs have fulfilled this requirement in the performed dissolution study. At elevated pH, 6.8, at least 75% of the drug should be released after 2.5 h for a dosage of 20 mg or 70% for 40 mg. Again, both drugs met the specification criteria.

HDPE bottles and aluminum foil blisters were used for the packaging closure system to evaluate the protective effect of the packaging materials on the developed product quality characteristics. According to the ICH guidance on stability testing, the studied batches had the same formulation and were packaged in the same container closure system proposed for the market [14]. The original drug Losec^®^ had a shelf life of 24 months. The aim was to reach the same term or longer for the developed generic formulation. For long-term studies, testing frequency was established every three months over the first year, every six months over the second year, and annually until the end of the specification. At the accelerated storage condition, more frequent time points (0, 1, 2, 3, and 6 months from a 6-month study) were determined to analyze the trend of the change in quality characteristics [14,15]. During the accelerated six-month and long-term twelve-month studies, the developed formulation in both packaging materials at each control point passed the average weight and mass uniformity test, dissolution test, acid-resistance stage test, buffer stage test, impurity assay, and microbiological purity test and met all the specification criteria. The results of the stability studies were considered sufficient at the time of the marketing authorization submission. They had been aimed to be continued for a time, covering the proposed shelf life of 24 months.

Achieving compliance with the criteria of the dissolution test in the acid resistance stage and the buffer stage and confirmation of product stability allowed the evaluation of the generic formulation in the BES. Based on the results of validation, it was established that the developed method of HPLC with tandem mass spectrometry can be used in pharmacokinetic and toxicokinetic studies in all phases of clinical trials, including studies of comparative pharmacokinetics and bioequivalence of drugs containing omeprazole. Similar analytical systems have been used successfully in other bioequivalence studies with omeprazole [22,23]. The clinical part of the study included the preparation of the BES, selection of volunteers, control of the intake of study drugs, blood sampling, and preparation of plasma for further bioanalytical study. A bioequivalence assessment focuses on a comparison of the drug absorption rates and extents of two formulations, not the therapeutic effectiveness of a formulation [24]. For this reason, a control or placebo group was not included in the study. According to the EMEA Guideline on the investigation of bioequivalence, the subject population for bioequivalence studies should be selected with the aim of permitting the detection of differences between pharmaceutical products [24]. Reducing subject variability is recommended; thus, a bioequivalence study does not monitor pharmacokinetic differences among different subject groups. Subjects should be, for example, healthy volunteers, 18 years of age or older, and preferably have a Body Mass Index between 18.5 and 30 kg/m^2^. These recommendations regarding subject selection were followed in the study, similar to another bioequivalence study with omeprazole [22]. During the BES, no adverse events were observed. Drug–drug and drug–disease interactions were not evaluated. The values of pharmacokinetic parameters AUC_0-t_ and C_max_ found in this study were approximately twice as large as those in a bioequivalence study with half-dose omeprazole [22]. Based on the data obtained as part of the analysis of variance, 90% confidence intervals were constructed [25,26,27]. The conclusion about the bioequivalence of the compared drugs was made using an approach based on the assessment of 90% confidence intervals for the ratios of the mean values of the logarithmically transformed pharmacokinetic parameters AUC_0-t_, C_max_, and C_max_/AUC_0-t_. The limits for AUC and C_max_ are in the range of 80–125%. For C_max_/AUC values, which were characterized by more significant variability, these limits are 75.00–133.00% [16]. A comparative analysis of the pharmacokinetic parameters of omeprazole, which was a part of the study drug, showed that the test drug omeprazole Viva Pharm, enteric capsules, 40 mg, is bioequivalent to the reference drug Losec^®^, enteric capsules, 20 mg (two capsules).

## 5. Conclusions

The quantitative and qualitative composition and technology for producing a new generic enteric form of omeprazole in capsules were developed and implemented at the solid-dosage-form production facility of the company Viva Pharm LLP, located in Kazakhstan. The technological parameters of the production process were established and specified in production protocols for commercialized product batches. The developed drug in two types of packaging has passed the stability test. An analytical normative document consisting of full quality control test methods was created for the experimental part and the registration dossier. The equivalence of the generic formulation omeprazole Viva Pharm, enteric capsules, to the innovative drug Losec^®^, enteric capsules, was proved by the dissolution profile test and BES. The new generic drug is aimed to be commercialized in the pharmaceutical market of the Republic of Kazakhstan after obtaining marketing authorization. Similarly to Europe, Kazakhstan relies on the localization of pharmaceutical drug production as one of its primary strategies to ensure that its population has access to affordable and high-quality medicines.

## Figures and Tables

**Table 1:** Schedule of the bioequivalence study.

Period of Study/Procedure	Screening	Study Periods			Final Examination
		I	Washout Period	II	
Thermometry (temperature should be between 36.0° and 36.6°)	Before screening	Before period I	Seven days	Before period II	
Duration of period	up to 10 days	13 h		13 h	
Informed consent	X				
Inclusion criteria	X	X		X	
Non-inclusion criteria	X	X		X	
Demographic and anthropometric data	X				
Medical history	X				
Physical examination, assessment of vital signs (blood pressure, heart rate, body temperature)	X	X		X	X
Laboratory examination (clinical blood test, biochemical blood test, general urine test)	X				X
Laboratory examination (clinical blood test, biochemical blood test, general urine test)	X				
Urine pregnancy test	X	X		X	
Urine analysis for psychotropic and narcotic substances, psychoactive drugs	X	X		X	
Alcohol test (breathalyzer/test strips)	X	X		X	
Hospitalization		X		X	
Electrocardiogram	X				X
Randomization		X			
Administration of T or R drug		X		X	
Blood sampling for PC		X		X	
Assessment of A.E.s/SAEs	X	X	X	X	X

**Table 2:** Chronology of composition changes (per capsule).

Component	Function	Composition 1 (%)	Composition 2 (%)	Composition 3 (%)	Composition 4 (%)
Microcrystalline cellulose (MCC) pellets	Neutral cores	60.45	64.8	62.75	62.44
Omeprazole	API	10.91	9.09	8.81	8.76
Lactose monohydrate	Filler	9.28	7.73	7.48	7.44
Sodium lauryl sulfate	Solubilizer	0.33	0.27	0.26	0.26
Sodium pyrophosphate dodecahydrate	Buffer agent	1.42	1.18	1.14	1.14
Hydrohypropylmethyl- cellulose	Film-former/ Binder	7.97	8.55	8.27	8.67
Hydrohypropylcellulose	Film-former/ Binder	2.18	0.91	0.88	0.44
Eudragit L30-D55	Enteric coating agent	5.76	6.29	8.79	8.75
PlasAcryl	Anti-tacking /Plasticizer	-	1.07	1.5	1.49
Polyethylene glycol		1.6	-	-	-
Titanium dioxide	Colorant	-	-	-	0.44
Talc	Lubricant	0.1	0.11	0,12	0,18
Purified water ^1^	Solvent	+	+	+	+
**Total capsule content weight, %**		**100**	**100**	**100**	**100**
**Weight of capsule content: 20 mg**		**218.0**	**220.0**	**227.27**	**228.4**
**Weight of capsule content: 40 mg**		**436.0**	**440.0**	**454.54**	**456.8**
**Capsule shell—body and cap composition, %:**					
Titanium dioxide	Colorant	2.0	2.0	2.0	2.0
Gelatin	Base	up to 100	up to 100	up to 100	up to 100

^1^ Not contained in the finished product.

**Table 3:** The average pharmacokinetic parameters and relative bioavailability of omeprazole for the test and reference products.

Group	Parameter	T1/2, h	tmax, h	Cmax, ng/mL	AUC_0-t_, ng/mL·h	AUC_0-∞_ ng/mL·h	(AUC0-t/_AUC0-∞_), %	Cmax/AUC_0-t_, h^−1^	Cmax/AUC_0-∞_, h^−1^	kel
**Test**	Arithmetic mean	1.83	2.54	1320.578	4520.85	4635.99	97.347	0.2936	0.2856	0.3819
	Geometric mean	1.82	2.50	1299.250	4448.57	4571.08	97.320	0.2921	0.2842	0.3799
	Median	1.84	2.50	1304.045	4416.58	4496.93	98.164	0.2878	0.2803	0.3776
	Minimum	1.42	2.00	945.489	2880.87	3044.90	89.859	0.2415	0.2390	0.3341
	Maximum	2.07	3.00	1984.091	6454.10	6571.64	98.969	0.3503	0.3374	0.4897
	Standard deviation	0.18	0.44	249.011	840.65	814.21	2.295	0.0311	0.0292	0.0415
	CV, %	9.8	17.3	18.9	18.6	17.6	2.4	10.6	10.2	10.9
**Reference**	Arithmetic mean	1.82	2.44	1273.837	4370.70	4502.17	96.841	0.2914	0.2821	0.3844
	Geometric mean	1.81	2.40	1253.036	4313.69	4456.15	96.803	0.2905	0.2812	0.3824
	Median	1.85	2.50	1284.560	4491.26	4568.94	98.192	0.2932	0.2878	0.3751
	Minimum	1.44	2.00	782.761	2773.41	2988.49	89.755	0.2546	0.2509	0.3145
	Maximum	2.20	3.00	1789.898	5566.99	5650.57	98.532	0.3403	0.3244	0.4810
	Standard deviation	0.18	0.43	233.311	695.23	640.08	2.742	0.0233	0.0226	0.0418
	CV, %	9.9	17.4	18.3	15.9	14.2	2.8	8.0	8.0	10.9

**Table 4:** The criteria for the observation period sufficiency.

Drug	Geometric Mean Values of Pharmacokinetic Parameters		AUC_0-t_/ AUC_0-∞_, %	Criteria for the Observation Period Sufficiency: AUC_0-t_ > 80% × AUC_0-∞_
	AUC_0-t_, ng/mL·h	AUC_0-∞_, ng/mL·h		
Test	4448.57	4571.08	97.3	Completed
Reference	4313.69	4456.15	96.8	Completed

**Table 5:** Assessment of BES statistical test power for test and reference.

PK Parameter	MSE_W_ (σ^2^)	CV_intra_	CV_intra_ (%)	Significance Level	Group Sizes	Power
ln(C_max_)	0.03105	0.1776	17.76	α = 5%	(12, 12)	99.9
ln(AUC_0→t_)	0.02067	0.1445	14.45			100.0
ln(C_max_/_AUC0→t_)	0.00659	0.0813	8.13			100.0

## Data Availability

The data presented in this study are available in the article.

## Supplementary Materials

The following supporting information can be downloaded at https://www.mdpi.com/article/10.3390/medicina60030427/s1: Subject inclusion criteria; Criteria for non-inclusion of subjects; Criteria for stopping subjects from participating in the study; Table S1: Comparative assessment of the release kinetics of omeprazole, dosage 20 mg; Table S2: Comparative assessment of the release kinetics of omeprazole, dosage 40 m; Table S3: Long-term stability study results of Omeprazole Viva Pharm, enteric capsules, 20 mg and 40 mg (batch size: 45.68 kg size/200,000 caps. (20 mg)/100,000 caps. (40 mg). Conditions: Temperature: 25 °C ± 2 °C, and the relative humidity (RH) 60% ± 5%; Table S4: Accelerated stability study results of Omeprazole Viva Pharm, enteric capsules, 20 mg and 40 mg (batch size: 45.68 kg size/200,000 caps. (20 mg)/100,000 caps. (40 mg). Conditions: Temperature: 40 °C ± 2 °C, and the relative humidity (RH) 75% ± 5%; Table S5: Concentrations of omeprazole in the blood plasma of volunteers (ng/mL) after a single dose of the drug Omeprazole (test drug “T”); Table S6: Concentrations of omeprazole in the blood plasma of volunteers (ng/mL) after a single dose of Losec^®^ (reference drug “R”); Table S7: Pharmacokinetic parameters of omeprazole in the blood plasma of volunteers after a single dose of the drug Omeprazole (test drug “T”); Table S8: Pharmacokinetic parameters of omeprazole in the blood plasma of volunteers after a single dose of Losec^®^ (reference drug “R”).
