# Supplementary material for: Development of a New Bioequivalent Omeprazole Product
Source: Medicina (Kaunas). 2024 Mar 2;60(3):427. doi: 10.3390/medicina60030427 (PMC10972143; doi:10.3390/medicina60030427)
Supplement: Supplementary file 1 [file medicina-60-00427-s001.zip › medicina-2817489-supplementary.pdf]

Subject inclusion criteria:
• men and women aged 18-45 years inclusive;
• body mass index (BMI) is within normal limits (≥18.5 kg/m2 and ≤30 kg/m2);
• verified diagnosis "healthy": absence of pathology from the gastrointestinal tract, liver, kidneys, cardiovascular system, central nervous system, endocrine system (preliminary standard clinical, laboratory and instrumental studies did not reveal the presence of any diseases; no deviations from standards based on the results of laboratory and instrumental examination methods, including ECG);
• for women, a negative pregnancy test. Women of childbearing age and male participants use barrier methods of contraception. If hormonal contraceptives are used, they should be discontinued while participating in the study;
• availability of written informed consent from the volunteer to participate in the study;
• thermometry (temperature should be from 36.0º to 36.6º).

Criteria for non-inclusion of subjects:
• thermometry (temperature should be from 36.0º to 36.6º);
• complicated allergy history, drug intolerance;
• hypersensitivity to Omeprazole and other components of the drug;
• galactose intolerance, lactase deficiency, glucose-galactose deficiency;
• systolic blood pressure less than 100 mm Hg. and more than 130 mm Hg; diastolic blood pressure less than 70 mm Hg. and more than 90 mm Hg; Heart rate is less than 60 beats/min and more than 90 beats/min at rest;
• surgical interventions on the gastrointestinal tract (with the exception of appendectomy);
• acute infectious diseases (influenza) less than 4 weeks before the start of the study;
• regular intake of medications (including vitamins, dietary supplements, medicinal herbs) less than 2 weeks before the start of the study;
• taking medications that have a pronounced effect on hemodynamics, liver function, etc. (barbiturates, omeprazole, cimetidine, etc.) less than 30 days before the start of the study;
• donation (450 ml of blood or plasma or more) less than 2 months before the start of the study;
• patients with chronic alcohol consumption (within the last 2 years), severe gastrointestinal, kidney, liver or cardiovascular diseases, tuberculous epilepsy, asthma (within 5 years), diabetes, psychosis or glaucoma;
• smoking more than 10 cigarettes per day;
• participation in a clinical trial of drugs less than 3 months before the start of the study;
• positive test for drugs and alcohol;
• positive test for HIV, Hepatitis B and C and syphilis (microreaction);
• pregnancy and breastfeeding period.

Criteria for stopping subjects from participating in the study:
The Principal Investigator may remove a volunteer from the study at any time if:
• the researcher decided that the volunteer should be excluded in the interests of the volunteer himself;

• adverse events have occurred that, due to their severity, make further participation of the volunteer in the study impossible;
• refusal on the part of the volunteer to follow the requirements of the protocol and carry out research procedures, inclusion of a volunteer in violation of the rules of the protocol (research error);
• the volunteer receives/needs additional treatment that may affect the pharmacokinetics of the study drug;
• the volunteer requires hospital treatment during the study;
• volunteers arrive at the clinic later than the appointed time (more than 1 hour late), which violates the protocol requirements;
• if the volunteer’s health deteriorates;
• positive test for drug abuse and drug use;
• positive breath alcohol test;
• positive pregnancy test.

Table S1. Comparative assessment of the release kinetics of omeprazole, dosage 20 mg.

No.	Time, min	Test drug		Reference drug	
		Release, average, %	RSD, %	Release, average, %	RSD, %
		**Acid resistance stage in pH 1.2**			
1.	120	8,68	0,64	3,27	2,38
		Buffer stage in pH 6.8			
2.	0	0	0	0	0
3.	10	84,53	10,10	90,16	4,00
4.	15	90,22	8,44	100,16	2,27
5.	20	91,35	6,96	100,78	1,07
6.	30	93,07	5,97	101,72	1,47
7.	45	93,97	3,97	100,41	1,31

Table S2. Comparative assessment of the release kinetics of omeprazole, dosage 40 mg.

No.	Time, min	Test drug		Reference drug	
		Release, average, %	RSD, %	Release, average, %	RSD, %
		**Acid resistance stage in pH 1.2**			
1.	120	11,90	7,78	13,82	17,66
		Buffer stage in pH 6.8			
2.	0	0	0	0	0
3.	10	86,34	3,94	68,64	2,70
4.	15	88,12	3,42	81,09	0,79
5.	20	90,10	1,45	85,18	1,36

[illegible].	30	88,90	1,57	86,69	1,47
7.	45	87,11	1,42	88,88	1,84
	f2		50,03		

Table S3. Long-term stability study results of Omeprazole Viva Pharm, enteric capsules, 20 mg and 40 mg (batch size: 45.68 kg size/200 000 caps. (20 mg)/100 000 caps. (40 mg). Conditions: Temperature: 25°C ± 2°C, and the relative humidity (RH) 60% ± 5%.

Test	Method	Specification	Dosage	Type of package	Results/Months				
					0	3	6	9	12
**Appearance**	Visual Inspection	Hard gelatin, cylindrical capsules no. 2 (for dosage 20 mg) and no.0 (for dosage 40 mg), with a cap and body of white or almost white color.<br>The contents of the capsules are a mixture of white or off-white pellets with a cream tint.	20 mg capsules	HDP bottles	Conforms	Conforms	Conforms	Conforms	Conforms
				Alu/Alu blisters	Conforms	Conforms	Conforms	Conforms	Conforms
			40 mg capsules	HDP bottles	Conforms	Conforms	Conforms	Conforms	Conforms
				Alu/Alu blisters	Conforms	Conforms	Conforms	Conforms	Conforms
**Average of capsule contents mass and Uniformity of mass**	Weighing	Deviation from the average weight is allowed for 18 capsules out of 20 no more than ± 10.0% and for 2 capsules out of 20 no more than ± 20% (for dosage 20 mg).	20 mg capsules	HDP bottles	234,1 mg 20/20 + 2,3 %: - 2,3 %	235,0 mg 20/20 - 5,0 %: + 2,9 %	234,9 mg 20/20 -4,1%: +3,7%	229,0 mg; 19/20 +7,6%; -4,2% 1/20: - 12,0%	234,8 mg 20/20 -4,6%: +3,8%
				Alu/Alu blisters	235,1 mg 20/20 + 3,3%: - 4,1%	234,8 mg 20/20 - 2,0%: + 2,7%	234,7 mg 20/20 -3,1%: +4,2%	234,9mg 20/20 -5,1%: +3,8%	235,0 mg 20/20 -4,2%: +4,8%
		Deviation from the average weight is allowed for 18 capsules out of 20 no more than ± 7.5% and for 2 capsules out of 20 no more than ± 15% (for dosage 40 mg).	40 mg capsules	HDP bottles	458,5 mg 20/20 + 1,0%; - 1,2%	460,2 mg 20/20 - 2,2%; +1,4%	459,3 mg 20/20 -1,2%; +1,9%	457,2 mg 20/20 -1,9%; +1,8%	459,7 mg 20/20 -1,0%; +1,7%
				Alu/Alu blisters	459,6 mg 20/20 + 1,7%; - 1,7%	458,2 mg 20/20 - 1,2%; +1,6%	459,1 mg 20/20 -2,0%; +1,9%	456,3 mg 20/20 -1,6%; +2,0%	458,7 mg 20/20 -1,0%; +1,3%

**Dissolution**<br>Acid Resistance stage	HPLC	No more than 15 % after 2 ч	20 mg capsules	HDP bottles	from 7,5 to 14,6 %	from 3,6 to 5,4 %	from 3,6 to 5,4%	from 3,9 to 6,4%	from 4,2 to 6,5%
				Alu/Alu blisters	from 5,5 to 7,8 %	from 5,9 to 8,3 %	from 3,7 to 5,4%	from 4,8 to 7,2%	from 4,8 to 6,9%
			40 mg capsules	HDP bottles	from 7,6 to 11,8 %	from 8,4 to 12,4 %	from 7,6 to 11,9%	from 7,7 to 11,0%	from 7,0 to 11,2%
				Alu/Alu blisters	from 7,5 to 10,8 %	from 7,9 to 11,3 %	from 7,7 to 10,4%	from 7,8 to 11,2%	from 7,8 to 10,9%
**Dissolution**<br>Buffer Stage	HPLC	No less than 75 % (for dosage 20 mg) and 70 % (for dosage 40 mg) after 2,5 ч	20 mg capsules	HDP bottles	from 75,6 to 78,3%	from 99,5 to 103,4%	from 85,6 to 98,2%	from 99,2 to 101,4%	from 88,6 to 99,1%
				Alu/Alu blisters	from 80,9 to 91,3 %	from 86,5 to 101,4 %	from 86,5 to 101,4%	from 88,5 to 99.3 %	from 91,2 to 100,1%
			40 mg capsules	HDP bottles	from 85,6 to 93,3%	from 89,9 to 99,8%	from 75,7 to 99,8%	from 88,2 to 100,0%	from 77,7 to 95,1%
				Alu/Alu blisters	from 75,9 to 89,3%	from 89,9 to 100.9%	from 75,9 to 95,2%	from 76,2 to 93,0%	from 88,0 to 95,1%
**Impurities:**<br>- sum of impurities F and G<br>- 5-methoxy-1H-benzimidazole-2-thiol<br>- any other impurity<br>- total impurities	HPLC	≤ 0,5%<br>≤ 0,5 %<br>≤ 0,5 %<br>≤ 2,0 %	20 mg capsules	HDP bottles	not detected<br>not detected<br>0,1%<br>0,1%	0,17%<br>0,18%<br>0,09%<br>0,14%	0,16%<br>not detected<br>0,09%<br>0,23%	0,19%<br>0,04%<br>0,12%<br>0,08%	0,18%<br>0,1%<br>0,12%<br>0,15%
				Alu/Alu blisters	not detected<br>not detected<br>not detected<br>not detected	0,05 %<br>not detected<br>0,04%<br>0,1%	0,06%<br>0,02%<br>not detected<br>0,08%	0,06%<br>0,03%<br>0,04%<br>0,1%	0,05%<br>0,1%<br>0,09%<br>0,11%
			40 mg capsules	HDP bottles	0,1%<br>not detected<br>not detected<br>not detected	0,05%<br>not detected<br>not detected<br>not detected	0,1%<br>0,08%<br>0,09%<br>0,12%	0,1%<br>not detected<br>0,11%<br>0,09%	0,15%<br>0,08%<br>0,1%<br>0,1%
				Alu/Alu blisters	not detected<br>not detected<br>not detected<br>not detected	0,04%<br>0,05%<br>not detected<br>not detected	0,05%<br>0,05%<br>0,04%<br>not detected	0,1%<br>0,03%<br>0,05%<br>0,1%	0,1%<br>0,08%<br>0,1%<br>0,11%
**Assay:**	HPLC			HDP bottles	20,9	19,7	21,5	21,5	21,0

- omeprazole		From 18,0 to 22,0 mg/caps (for dosage 20 mg)<br>From 36,0 to 44,0 mg/caps (for dosage 20 mg)	20 mg capsules	Alu/Alu blisters	19,9	19,9	21,3	21,0	20,8
			40 mg capsules	HDP bottles	39,3	40,2	41,4	39,2	39,9
				Alu/Alu blisters	38,1	41,5	40,9	40,9	39,2
**Total Aerobic Microbial Count (TAMC),** cfu/1 g	Direct	≤ 10^3	20 mg capsules	HDP bottles	< 10 cfu/g	< 10 cfu/g	< 10 cfu/g	< 10 cfu/g	< 10 cfu/g
				Alu/Alu blisters	< 10 cfu/g	< 10 cfu/g	< 10 cfu/g	< 10 cfu/g	< 10 cfu/g
			40 mg capsules	HDP bottles	< 10 cfu/g	< 10 cfu/g	< 10 cfu/g	< 10 cfu/g	< 10 cfu/g
				Alu/Alu blisters	< 10 cfu/g	< 10 cfu/g	< 10 cfu/g	< 10 cfu/g	< 10 cfu/g
**Total Combined Yeasts and Moulds Count,** cfu/1 g	Direct	≤ 10^2	20 mg capsules	HDP bottles	< 10 cfu/g	< 10 cfu/g	< 10 cfu/g	< 10 cfu/g	< 10 cfu/g
				Alu/Alu blisters	< 10 cfu/g	< 10 cfu/g	< 10 cfu/g	< 10 cfu/g	< 10 cfu/g
			40 mg capsules	HDP bottles	< 10 cfu/g	< 10 cfu/g	< 10 cfu/g	< 10 cfu/g	< 10 cfu/g
				Alu/Alu blisters	< 10 cfu/g	< 10 cfu/g	< 10 cfu/g	< 10 cfu/g	< 10 cfu/g
**Escherichia coli** in 1 g	Direct	Absence in 1 g	20 mg capsules	HDP bottles	not detected	not detected	not detected	not detected	not detected
				Alu/Alu blisters	not detected	not detected	not detected	not detected	not detected
			40 mg capsules	HDP bottles	not detected	not detected	not detected	not detected	not detected
				Alu/Alu blisters	not detected	not detected	not detected	not detected	not detected

Table S4. Accelerated stability study results of Omeprazole Viva Pharm, enteric capsules, 20 mg and 40 mg (batch size: 45.68 kg size/200 000 caps. (20 mg)/100 000 caps. (40 mg). Conditions: Temperature: 40°C ± 2°C, and the relative humidity (RH) 75% ± 5%.

**Test**	**Method**	**Specification**	**Dosage**	**Type of package**	**Results/Months**				
					**0**	**1**	**2**	**3**	**6**
**Appearance**	Visual Inspection	Hard gelatin, cylindrical capsules no. 2 (for dosage 20 mg) and no.0 (for dosage 40 mg), with a cap and body of white or almost white color. The contents of the capsules are a mixture of white or off-white pellets with a cream tint.	20 mg capsules	HDP bottles	Conforms	Conforms	Conforms	Conforms	Conforms
				Alu/Alu blisters	Conforms	Conforms	Conforms	Conforms	Conforms
			40 mg capsules	HDP bottles	Conforms	Conforms	Conforms	Conforms	Conforms
				Alu/Alu blisters	Conforms	Conforms	Conforms	Conforms	Conforms
**Average of capsule contents mass and Uniformity of mass**	Weighing	Deviation from the average weight is allowed for 18 capsules out of 20 no more than ± 10.0% and for 2 capsules out of 20 no more than ± 20% (for dosage 20 mg). Deviation from the average weight is allowed for 18 capsules out of 20 no more than ± 7.5% and for 2 capsules out of 20 no more than ± 15% (for dosage 40 mg).	20 mg capsules	HDP bottles	235,2 mg 20/20 + 4,3 %: - 2,8 %	235,2 mg 20/20 - 2,3 %: + 2,8 %	232,8 mg 20/20 -5,2%: +4,1%	230,0 mg; 19/20 +6,6%; -5,3% 1/20: - 12,0%	229,0 mg; 19/20 +7,6%; -4,2% 1/20: - 12,0%
				Alu/Alu blisters	239,1 mg 20/20 + 2,3%: - 428%	239,8 mg 20/20 - 3,0%: + 2,9%	238,6 mg 20/20 -3,8%: +5,1%	239,9mg 20/20 -3,1%: +4,1%	238,0 mg 20/20 -2,2%: +4,6%
			40 mg capsules	HDP bottles	459,0 mg 20/20 + 2,0%; - 2,7%	459,2 mg 20/20 - 2,2%; +2,7%	457,7 mg 20/20 -2,2%; +1,9%	458,5 mg 20/20 -1,9%; +1,9%	458,9 mg 20/20 -2,1%; +2,8%
				Alu/Alu blisters	460,1 mg 20/20: + 1,9%; - 1,3%	459,0 mg 20/20 - 1,3%; +1,6%	457,3 mg 20/20 -1,9%; +2,8%	457,9 mg 20/20 -1,8%; +1,0%	458,6 mg 20/20 -1,8%; +1,3%
**Dissolution** Acid Resistance stage	HPLC	No more than 15 % after 2 ч	20 mg capsules	HDP bottles	from 7,7 to 10,9 %	from 5,5 to 7,1 %	from 4,0 to 5,3%	from 3,8 to 6,4%	from 4,9 to 6,8%
				Alu/Alu blisters	from 5,5 to 6,9 %	from 6,0 to 7,1 %	from 4,7 to 6,5%	from 5,5 to 7,0%	from 4,0 to 6,0%

			40 mg capsules	HDP bottles	from 7,7 to 11,1 %	from 7,3 to 12,0 %	from 6,6 to 10,0%	from 7,1 to 12,0%	from 7,7 to 10,3%
				Alu/Alu blisters	from 7,8 to 11,8 %	from 6,9 to 10,2 %	from 7,6 to 9,9%	from 6,8 to 11,5%	from 7,5 to 11,2%
**Dissolution**<br>Buffer Stage	HPLC	No less than 75 % (for dosage 20 mg) and 70 % (for dosage 40 mg) after 2,5 ч	20 mg capsules	HDP bottles	from 77,7 to 79,6%	from 88,7 to 99,8%	from 89,0 to 101,1%	from 88,0 to 99,3%	from 88,8 to 99,1%
				Alu/Alu blisters	from 80,9 to 92,7 %	from 88,5 to 100,0 %	from 86,5 to 101,2%	from 86,0 to 99.2 %	from 90,2 to 99,0%
			40 mg capsules	HDP bottles	from 76,6 to 91,7%	from 80,9 to 95,7%	from 79,7 to 96,6%	from 80,2 to 99,0%	from 79,9 to 96,9%
				Alu/Alu blisters	from 78,0 to 89,9%	from 79,9 to 101.0%	from 80,2 to 96,2%	from 79,5 to 95,0%	from 79,9 to 99,1%
**Impurities:**<br>- sum of impurities F and G<br>- 5-methoxy-1H-benzimidazole-2-thiol<br>- any other impurity<br>- total impurities	HPLC	≤ 0,5%<br>≤ 0,5 %<br>≤ 0,5 %<br>≤ 2,0 %	20 mg capsules	HDP bottles	not detected<br>not detected<br>not detected<br>not detected	0,19%<br>0,19%<br>0,09%<br>0,13%	0,16%<br>0,18%<br>0,1%<br>0,18%	0,1%<br>0,1%<br>0,12%<br>0,15%	0,19%<br>0,18%<br>0,15%<br>0,15%
				Alu/Alu blisters	not detected<br>0,05<br>not detected<br>0,08	0,05 %<br>0,05%<br>not detected<br>not detected	0,05%<br>0,03%<br>not detected<br>0,08%	0,06%<br>0,06%<br>0,04%<br>0,1%	0,06%<br>0,1%<br>0,08%<br>0,12%
			40 mg capsules	HDP bottles	0,1%<br>not detected<br>0,07<br>not detected	0,05%<br>not detected<br>not detected<br>not detected	0,1%<br>0,08%<br>0,09%<br>0,12%	0,1%<br>0,1%<br>0,15%<br>0,12%	0,15%<br>0,1%<br>0,15%<br>0,1%
				Alu/Alu blisters	not detected<br>not detected<br>not detected<br>not detected	0,05%<br>0,05%<br>0,05%<br>not detected	0,05%<br>0,1%<br>0,05%<br>not detected	0,1%<br>0,06%<br>0,1%<br>0,1%	0,1%<br>0,1%<br>0,1%<br>0,11%
**Assay:**<br>- omeprazole	HPLC	From 18,0 to 22,0 mg/caps (for dosage 20 mg)	20 mg capsules	HDP bottles	19,9	19,7	20,4	21,4	21,0
				Alu/Alu blisters	20,9	19,8	20,5	21,2	20,7
				HDP bottles	40,1	41,0	41,4	40,2	39,8

		From 36,0 to 44,0 mg/caps (for dosage 20 mg)	40 mg capsules	Alu/Alu blisters	39,2	40,5	39,9	40,2	40,2
**Total Aerobic Microbial Count (TAMC),** cfu/1 g	Direct	≤ 10^3	20 mg capsules	HDP bottles	< 10 cfu/g	< 10 cfu/g	< 10 cfu/g	< 10 cfu/g	< 10 cfu/g
				Alu/Alu blisters	< 10 cfu/g	< 10 cfu/g	< 10 cfu/g	< 10 cfu/g	< 10 cfu/g
			40 mg capsules	HDP bottles	< 10 cfu/g	< 10 cfu/g	< 10 cfu/g	< 10 cfu/g	< 10 cfu/g
				Alu/Alu blisters	< 10 cfu/g	< 10 cfu/g	< 10 cfu/g	< 10 cfu/g	< 10 cfu/g
**Total Combined Yeasts and Moulds Count,** cfu/1 g	Direct	≤ 10^2	20 mg capsules	HDP bottles	< 10 cfu/g	< 10 cfu/g	< 10 cfu/g	< 10 cfu/g	< 10 cfu/g
				Alu/Alu blisters	< 10 cfu/g	< 10 cfu/g	< 10 cfu/g	< 10 cfu/g	< 10 cfu/g
			40 mg capsules	HDP bottles	< 10 cfu/g	< 10 cfu/g	< 10 cfu/g	< 10 cfu/g	< 10 cfu/g
				Alu/Alu blisters	< 10 cfu/g	< 10 cfu/g	< 10 cfu/g	< 10 cfu/g	< 10 cfu/g
**Escherichia coli** in 1 g	Direct	Absence in 1 g	20 mg capsules	HDP bottles	not detected	not detected	not detected	not detected	not detected
				Alu/Alu blisters	not detected	not detected	not detected	not detected	not detected
			40 mg capsules	HDP bottles	not detected	not detected	not detected	not detected	not detected
				Alu/Alu blisters	not detected	not detected	not detected	not detected	not detected

Table S5. Concentrations of omeprazole in the blood plasma of volunteers (ng/ml) after a single dose of the drug Omeprazole (test drug “T”).

Time (t) after drug administration (planned), h	03	04	06	07	08	10	11	14	18	20	22	24	01	02	05	09	12	13	15	16	17	19	21	23	Mean	Gmean	Mediana	Minimum	Maximum	SD	CV
	I	I	I	I	I	I	I	I	I	I	I	I	II	II	II	II	II	II	II	II	II	II	II	II							
**0.00**	0.000	0.000	0.000	0.000	0.000	0.000	0.000	0.000	0.000	0.000	0.000	0.000	0.000	0.000	0.000	0.000	0.000	0.000	0.000	0.000	0.000	0.000	0.000	0.000	**0.000**	**0.000**	**0.000**	**0.000**	**0.000**	**0.000**	**-**
**0.50**	0.000	0.000	32.971	39.655	31.270	28.607	0.000	29.866	0.000	27.647	26.454	0.000	26.006	26.675	27.007	0.000	0.000	0.000	33.565	25.100	0.000	29.910	0.000	25.554	**17.095**	**29.073**	**25.780**	**0.000**	**39.655**	**15.064**	**88.1**
**1.00**	251.712	239.152	335.298	377.578	195.235	196.051	142.800	100.453	302.822	365.509	255.962	228.908	253.236	191.330	289.061	236.998	191.106	254.038	280.089	309.050	295.452	369.283	286.34	255.604	**258.462**	**248.324**	**254.821**	**100.453**	**377.578**	**68.843**	**26.6**
**1.50**	992.640	945.666	1316.184	1066.060	529.150	528.569	398.066	224.889	505.030	594.791	945.321	684.755	531.839	652.058	593.367	801.067	549.490	1052.695	968.463	809.200	434.367	519.217	501.646	939.260	**711.825**	**662.399**	**623.424**	**224.889**	**1316.184**	**265.750**	**37.3**
**2.00**	1218.614	1167.636	1597.860	1716.502	740.815	738.469	698.520	677.078	736.788	878.400	1143.976	1315.034	770.168	994.804	983.777	1227.525	947.000	1139.129	1492.415	1062.576	707.911	914.247	689.663	1140.286	**1029.133**	**990.330**	**989.291**	**677.078**	**1716.502**	**298.053**	**29.0**
**2.50**	1223.886	1147.988	1688.330	1734.934	1269.303	1264.715	945.489	1286.590	1050.205	1210.030	1038.824	1165.891	881.899	893.408	1235.663	1092.903	1095.674	1040.870	1154.744	1380.704	928.662	1314.989	906.571	1038.880	**1166.298**	**1148.121**	**1151.366**	**881.899**	**1734.934**	**219.617**	**18.8**
**3.00**	915.754	902.312	1426.038	1432.504	1401.148	1399.648	894.313	1352.892	1220.191	1405.422	868.549	1044.605	971.541	794.772	1293.055	968.862	903.269	865.395	956.263	987.947	1505.911	1984.091	1470.157	869.217	**1159.744**	**1125.293**	**1016.276**	**794.772**	**1984.091**	**300.946**	**25.9**
**4.00**	684.709	647.186	1088.830	1086.963	871.999	883.226	484.315	1112.968	740.169	856.626	687.993	797.992	729.851	532.514	782.677	655.790	507.074	658.790	665.974	545.027	986.931	1301.458	940.214	697.970	**789.469**	**763.616**	**735.010**	**484.315**	**1301.458**	**211.780**	**26.8**
**5.00**	358.638	353.766	511.004	701.817	464.526	368.952	237.565	771.882	329.296	374.586	458.838	474.966	445.981	434.510	500.833	533.805	378.517	468.954	480.228	387.047	368.577	487.146	349.530	465.883	**446.119**	**433.326**	**452.409**	**237.565**	**771.882**	**114.097**	**25.6**
**6.00**	248.584	236.572	353.426	341.990	266.492	279.976	212.185	456.730	263.904	299.009	321.674	280.676	210.000	236.734	212.194	290.203	244.949	326.651	230.601	225.632	231.063	308.116	221.323	330.033	**276.196**	**270.838**	**265.198**	**210.000**	**456.730**	**58.807**	**21.3**
**8.00**	126.511	122.095	184.605	175.873	140.769	143.230	77.541	268.062	103.275	117.543	115.506	123.879	105.906	136.511	120.564	169.149	93.598	113.424	140.301	136.226	104.730	141.435	98.727	117.291	**132.365**	**128.007**	**122.987**	**77.541**	**268.062**	**38.530**	**29.1**
**12.0**	28.774	28.545	42.327	41.486	28.590	25.312	0.000	26.863	27.388	30.931	31.333	27.312	0.000	0.000	27.813	27.850	28.243	27.763	25.952	0.000	30.367	39.978	28.922	32.376	**25.339**	**30.065**	**28.046**	**0.000**	**42.327**	**12.436**	**49.1**

Table S6. Concentrations of omeprazole in the blood plasma of volunteers (ng/ml) after a single dose of Losec® (reference drug "R").

Time (t) after drug administration (planned), h	01	02	05	09	12	13	15	16	17	19	21	23	03	04	06	07	08	10	11	14	18	20	22	24	Mean	Gmean	Mediana	Minimum	Maximum	SD	CV
	I	I	I	I	I	I	I	I	I	I	I	I	II	II	II	II	II	II	II	II	II	II	II	II							
0.00	0.000	0.000	0.000	0.000	0.000	0.000	0.000	0.000	0.000	0.000	0.000	0.000	0.000	0.000	0.000	0.000	0.000	0.000	0.000	0.000	0.000	0.000	0.000	0.000	**0.000**	**-**	**0.000**	**0.000**	**0.000**	**0.000**	**-**
0.50	31.623	28.507	30.389	29.518	25.284	0.000	0.000	0.000	26.934	0.000	0.000	0.000	0.000	0.000	27.188	0.000	0.000	0.000	0.000	0.000	44.680	39.640	31.684	33.107	**14.523**	**31.256**	**0.000**	**0.000**	**44.680**	**16.572**	**114.1**
1.00	217.944	279.541	225.365	221.942	237.435	262.513	241.655	209.829	278.455	341.168	307.071	228.244	184.154	200.758	263.287	281.593	279.651	292.748	193.578	247.877	332.190	413.725	330.300	354.387	**267.725**	**262.161**	**262.900**	**184.154**	**413.725**	**57.233**	**21.4**
1.50	862.526	1094.910	897.765	885.881	689.147	1069.999	985.275	636.179	933.500	573.383	511.818	674.184	604.581	424.628	904.271	601.901	579.299	612.259	557.921	1012.103	1132.124	1067.719	880.643	913.513	**796.064**	**767.491**	**871.584**	**424.628**	**1132.124**	**212.641**	**26.7**
2.00	1301.387	1343.552	1159.069	1327.830	817.410	1163.493	1066.764	1187.371	1221.420	1075.465	739.502	1288.543	926.910	590.397	1375.391	866.924	742.674	1007.402	933.018	1101.525	1698.926	1404.304	1153.976	1183.633	**1111.537**	**1082.035**	**1156.522**	**590.397**	**1698.926**	**252.874**	**22.7**
2.50	1201.919	1387.052	1431.989	1145.142	1146.325	1069.933	978.985	1280.577	1121.534	1215.542	1061.367	1151.065	831.391	697.287	1237.065	1015.503	999.300	1270.354	1055.676	1008.836	1352.077	1789.898	1500.225	1563.254	**1188.012**	**1165.069**	**1148.695**	**697.287**	**1789.898**	**238.776**	**20.1**
3.00	1067.688	941.309	1441.788	1017.964	839.120	867.118	859.859	1129.343	873.473	1440.801	1218.077	994.018	742.327	782.761	1106.697	1109.844	1068.367	1315.246	850.573	862.632	1130.949	1311.268	1077.753	1130.640	**1049.151**	**1031.637**	**1068.027**	**742.327**	**1441.788**	**198.122**	**18.9**
4.00	640.195	657.128	666.690	661.656	527.390	710.318	645.548	719.978	648.721	874.455	738.605	767.802	505.912	589.269	764.785	840.954	798.013	812.555	477.413	664.936	825.816	716.983	589.933	627.210	**686.344**	**678.269**	**665.813**	**477.413**	**874.455**	**105.607**	**15.4**
5.00	367.148	400.458	388.010	388.534	303.499	477.142	434.969	425.705	504.649	392.903	324.664	452.244	409.027	295.643	625.222	490.737	476.579	524.700	358.633	447.873	578.868	444.327	325.530	458.826	**428.995**	**421.531**	**430.337**	**295.643**	**625.222**	**82.368**	**19.2**
6.00	243.352	269.081	263.242	263.622	221.726	334.627	307.339	250.513	352.498	321.426	261.736	270.570	221.361	172.400	331.847	246.324	235.386	218.674	218.156	317.128	278.843	299.997	249.889	258.044	**266.991**	**263.437**	**262.489**	**172.400**	**352.498**	**44.195**	**16.6**
8.00	137.400	138.536	146.170	147.133	118.148	116.616	105.570	109.022	124.996	125.990	100.858	122.840	128.476	86.297	200.759	124.073	119.777	117.077	82.406	109.538	174.550	183.778	151.708	155.995	**130.321**	**127.430**	**124.534**	**82.406**	**200.759**	**28.675**	**22.0**
12.0	28.665	30.031	31.726	31.114	0.000	29.275	25.248	0.000	34.403	35.465	28.884	26.850	0.000	0.000	38.589	0.000	0.000	28.377	25.249	26.741	36.487	31.630	26.249	28.247	**22.635**	**29.950**	**28.312**	**0.000**	**38.589**	**13.767**	**60.8**

Table S7. Pharmacokinetic parameters of omeprazole in the blood plasma of volunteers after a single dose of the drug Omeprazole (test drug "T").

Randomized №	Period	$T_{1/2}$ h	t_{max} h	C_{max} ng/ml	$AUC_{0\to t}$ ng/ml×h	$AUC_{0\to\infty}$ ng/ml×h	AUMC ng/ml×h^2	MRT h	$C_{max}/AUC_{0\to t}$ h^{-1}	$C_{max}/AUC_{0\to\infty}$ h^{-1}
**01**	**II**	1.42	3.00	971.541	3556.97	3773.22	12477.07	3.51	0.2731	0.2575
**02**	**II**	1.97	2.00	994.804	3433.85	3821.40	12016.47	3.50	0.2897	0.2603
**03**	**I**	1.93	2.50	1223.886	4383.54	4463.54	16175.37	3.69	0.2792	0.2742
**04**	**I**	1.94	2.00	1167.636	4206.14	4286.13	15563.72	3.70	0.2776	0.2724
**05**	**II**	2.02	3.00	1293.055	4553.35	4634.51	17139.70	3.76	0.2840	0.2790
**06**	**I**	1.95	2.50	1688.330	6323.29	6442.65	23617.43	3.73	0.2670	0.2621
**07**	**I**	1.96	2.50	1734.934	6454.10	6571.64	24258.01	3.76	0.2688	0.2640
**08**	**I**	1.78	3.00	1401.148	4649.50	4723.05	18256.00	3.93	0.3014	0.2967
**09**	**II**	1.80	2.00	1227.525	4593.94	4666.20	18124.87	3.95	0.2672	0.2631
**10**	**I**	1.71	3.00	1399.648	4580.40	4642.86	17912.63	3.91	0.3056	0.3015
**11**	**I**	1.47	2.50	945.489	2880.87	3044.90	9917.61	3.44	0.3282	0.3105
**12**	**II**	1.81	2.50	1095.674	3659.38	3732.96	13906.22	3.80	0.2994	0.2935
**13**	**II**	1.78	2.00	1139.129	4405.93	4477.10	16612.19	3.77	0.2585	0.2544
**14**	**I**	1.51	3.00	1352.892	5601.97	5660.30	25055.75	4.47	0.2415	0.2390
**15**	**II**	1.73	2.00	1492.415	4646.75	4711.56	17092.75	3.68	0.3212	0.3168
**16**	**II**	1.76	2.50	1380.704	3941.02	4286.97	13014.88	3.30	0.3503	0.3221
**17**	**II**	2.07	3.00	1505.911	4389.66	4480.54	16843.04	3.84	0.3431	0.3361
**18**	**I**	1.90	3.00	1220.191	4042.49	4117.68	15443.35	3.82	0.3018	0.2963
**19**	**II**	2.06	3.00	1984.091	5816.93	5935.61	22372.25	3.85	0.3411	0.3343
**20**	**I**	1.90	3.00	1405.422	4686.47	4771.20	17742.79	3.79	0.2999	0.2946
**21**	**II**	2.07	3.00	1470.157	4270.49	4356.94	16214.14	3.80	0.3443	0.3374
**22**	**I**	1.83	2.00	1143.976	4395.21	4477.85	16705.70	3.80	0.2603	0.2555
**23**	**II**	1.84	2.00	1140.286	4427.23	4513.33	16916.86	3.82	0.2576	0.2526
**24**	**I**	1.79	2.00	1315.034	4600.98	4671.58	17472.25	3.80	0.2858	0.2815
**Mean**		**1.83**	**2.54**	**1320.578**	**4520.85**	**4635.99**	**17118.79**	**3.77**	**0.2936**	**0.2856**
**Gmean**		**1.82**	**2.50**	**1299.250**	**4448.57**	**4571.08**	**16732.26**	**3.76**	**0.2921**	**0.2842**

**Mediana**		**1.84**	**2.50**	**1304.045**	**4416.58**	**4496.93**	**16879.95**	**3.79**	**0.2878**	**0.2803**
**Minimum**		**1.42**	**2.00**	**945.489**	**2880.87**	**3044.90**	**9917.61**	**3.30**	**0.2415**	**0.2390**
**Maximum**		**2.07**	**3.00**	**1984.091**	**6454.10**	**6571.64**	**25055.75**	**4.47**	**0.3503**	**0.3374**
**SD**		**0.18**	**0.44**	**249.011**	**840.65**	**814.21**	**3737.72**	**0.22**	**0.0311**	**0.0292**
**CV**		**9.8**	**17.3**	**18.9**	**18.6**	**17.6**	**21.8**	**5.7**	**10.6**	**10.2**

Table S8. Pharmacokinetic parameters of omeprazole in the blood plasma of volunteers after a single dose of Losec® (reference drug "R").

**Randomized №**	**Period**	**$T_{1/2}$ h**	**t_{max} h**	**C_{max} ng/ml**	**$AUC_{0\to t}$ ng/ml×h**	**$AUC_{0\to\infty}$ ng/ml×h**	**AUMC ng/ml×h²**	**MRT h**	**$C_{max}/AUC_{0\to t}$ h^{-1}**	**$C_{max}/AUC_{0\to\infty}$ h^{-1}**
**01**	**I**	1.92	2.00	1301.387	4450.37	4529.89	16542.11	3.72	0.2924	0.2873
**02**	**I**	1.88	2.50	1387.052	4709.64	4791.26	17261.53	3.67	0.2945	0.2895
**03**	**II**	1.97	2.00	926.910	3205.72	3571.63	11270.73	3.52	0.2891	0.2595
**04**	**II**	1.73	3.00	782.761	2773.41	2988.49	9719.34	3.50	0.2822	0.2619
**05**	**I**	1.95	3.00	1441.788	4905.15	4994.52	18232.18	3.72	0.2939	0.2887
**06**	**II**	1.89	2.00	1375.391	5300.86	5406.20	21144.66	3.99	0.2595	0.2544
**07**	**II**	1.44	3.00	1109.844	4040.59	4298.54	14252.68	3.53	0.2747	0.2582
**08**	**II**	1.45	3.00	1068.367	3849.18	4099.57	13621.56	3.54	0.2776	0.2606
**09**	**I**	1.94	2.00	1327.830	4517.88	4604.78	17072.25	3.78	0.2939	0.2884
**10**	**II**	2.02	3.00	1315.246	4651.07	4733.96	17492.91	3.76	0.2828	0.2778
**11**	**II**	1.77	2.50	1055.676	3469.02	3533.55	12931.56	3.73	0.3043	0.2988
**12**	**I**	2.20	2.50	1146.325	3368.77	3744.41	11348.00	3.37	0.3403	0.3061
**13**	**I**	1.71	2.00	1163.493	4531.11	4603.37	17125.68	3.78	0.2568	0.2527
**14**	**II**	1.76	2.00	1101.525	4302.74	4370.80	16210.47	3.77	0.2560	0.2520
**15**	**I**	1.71	2.00	1066.764	4189.97	4252.40	15747.69	3.76	0.2546	0.2509
**16**	**I**	1.46	2.50	1280.577	4134.46	4364.46	14046.39	3.40	0.3097	0.2934
**17**	**I**	1.87	2.00	1221.420	4571.94	4664.67	17550.91	3.84	0.2672	0.2618
**18**	**II**	1.81	2.00	1698.926	5547.78	5642.96	20732.10	3.74	0.3062	0.3011

**19**	**I**	1.96	3.00	1440.801	4881.78	4982.19	18667.42	3.82	0.2951	0.2892
**20**	**II**	1.83	2.50	1789.898	5566.99	5650.57	20568.93	3.69	0.3215	0.3168
**21**	**I**	1.95	3.00	1218.077	4039.65	4121.08	15391.46	3.81	0.3015	0.2956
**22**	**II**	1.86	2.50	1500.225	4554.65	4625.25	16795.10	3.69	0.3294	0.3244
**23**	**I**	1.80	2.00	1288.543	4464.65	4534.51	16943.78	3.80	0.2886	0.2842
**24**	**II**	1.80	2.50	1563.254	4869.51	4942.97	18099.42	3.72	0.3210	0.3163
**Mean**		**1.82**	**2.44**	**1273.837**	**4370.70**	**4502.17**	**16198.70**	**3.69**	**0.2914**	**0.2821**
**Gmean**		**1.81**	**2.40**	**1253.036**	**4313.69**	**4456.15**	**15915.59**	**3.69**	**0.2905**	**0.2812**
**Mediana**		**1.85**	**2.50**	**1284.560**	**4491.26**	**4568.94**	**16869.44**	**3.72**	**0.2932**	**0.2878**
**Minimum**		**1.44**	**2.00**	**782.761**	**2773.41**	**2988.49**	**9719.34**	**3.37**	**0.2546**	**0.2509**
**Maximum**		**2.20**	**3.00**	**1789.898**	**5566.99**	**5650.57**	**21144.66**	**3.99**	**0.3403**	**0.3244**
**SD**		**0.18**	**0.43**	**233.311**	**695.23**	**640.08**	**2960.81**	**0.15**	**0.0233**	**0.0226**
**CV**		**9.9**	**17.4**	**18.3**	**15.9**	**14.2**	**18.3**	**4.0**	**8.0**	**8.0**
